# Specific patterns of T-cell cytokines as an early marker of outcome in septic patients

**DOI:** 10.1186/cc14117

**Published:** 2015-03-16

**Authors:** A Franci, A Peris, F Liotta, F Annunziato, P Ruggiano, M Ferraro

**Affiliations:** 1 A.O.U. Careggi, Firenze, Italy

## Introduction

The inflammatory response of sepsis is developed in two phases, an inflammatory phase (SIRS) and a phase more variable in frequency and intensity (CARS): this balance has an important effect on morbidity and mortality. Lymphopenia affects particularly T cells, and correlates inversely with outcome. The aim of the study was to identify phenotypic and functional early markers of T cells and NK cells related to prognosis in the septic patient population.

## Methods

We collected peripheral blood mononuclear cells from 47 patients with severe sepsis or septic shock at ICU admission (T0) and from 50 healthy controls. On these subjects we evaluated frequency and absolute numbers of CD4^+ ^and CD8^+ ^T cells and of NK and B lymphocytes, the rates of regulatory CD4^+^CD25^+^Foxp3^+ ^T cells (Tregs), the cytotoxic potential of CD4^+^, CD8^+ ^T cells and of NK cells by evaluation of perforin (PER) and granzyme (GRA) expression and production of effector cytokines (namely IL-2, IL-17, IL-4, TNFα, IFNγ) by CD4^+^, CD8^+ ^T cells and NK cells upon polyclonal stimulation. The markers were compared in patients with different outcome.

## Results

Septic patients, compared with healthy donors, were characterized by global lymphopenia; we found increased frequencies of CD4^+ ^T cells producing IL-2 (*P *= 0.0000000003), increased percentage of CD8 T cells producing IFNγ (*P *= 0.03), and reduced proportion of CD4^+ ^T cells (*P *= 0.00007) and NK cells (*P *= 0.002) producing IFNγ. We also noticed an increased frequency of CD8^+ ^T cells expressing PER (*P *= 0.00000025) and GRA (*P *= 0.01); moreover, the proportion of NK cells expressing GRA was also significantly increased (*P *= 0.000019). To establish the prognostic value of these biological markers, we compared the cytokine expression by lymphocytes in septic patients that survived with those that died (D). We found that CD4^+ ^and CD8^+ ^TNFα-producing T cells were significantly increased in D (*P *= 0.01 and *P *= 0.0001 respectively); similarly the percentage of CD8^+ ^T cells producing IFNγ was more elevated in D (*P *= 0.006). The same was observed for IL-17 production by CD4^+ ^T cells (*P *= 0.03) in D. On the contrary we observed a tendency to the reduction of circulating CD4^+^CD25^+^foxp3 (Tregs) in D (*P *= 0.08).

## Conclusion

Septic patients are characterized by a peculiar immunophenotype which includes global lymphopenia and a specific pattern of cytokines. Some of the evaluated markers seem to individuate those with worse outcome; in particular, this group showed an inflammatory phenotype with a higher expression of IFNγ, TNFα, IL17 and a tendency to a reduction of Tregs.

